# A structured approach to developing an introductory statistics course for graduate students: Using data to teach about data

**DOI:** 10.1017/cts.2024.672

**Published:** 2024-12-16

**Authors:** Lisa Eunyoung Lee, Sobiga Vyravanathan, Tony Panzarella, Caitlin Gillan, Nicole Harnett

**Affiliations:** 1 Institute of Medical Science, University of Toronto, Toronto, ON, Canada; 2 Dalla Lana School of Public Health, University of Toronto, Toronto, ON, Canada; 3 Department of Radiation Oncology, University of Toronto, Toronto, ON, Canada

**Keywords:** Statistics, study design, course development, evaluation, medical science

## Abstract

**Background/Objective:**

It was identified in the largest graduate unit of the Faculty of Medicine of a major Canadian University that there was a critical unmet curricular need for an introductory statistics and study design course. Based on the collective findings of an external institute review, both quantitative and qualitative data were used to design, develop, implement, evaluate, and refine such a course.

**Methods:**

In response to the identified need and inherent challenges to streamlining curriculum development and instructional design in research-based graduate programs representing many biomedical disciplines, the institute used the analyze, design, develop, implement and evaluate instructional design model to guide the data-driven development and ongoing monitoring of a new study design and statistics course.

**Results:**

The results demonstrated that implementing recommendations from the first iteration of the course (Fall 2021) into the second iteration (Winter 2023) led to improved student learning experience (3.18/5 weighted average (Fall 2021) to 3.87/5 (Winter 2023)). In the second iteration of the course, a self-perceived statistics anxiety test was administered, showing a reduction in statistics anxiety levels after completing the course (2.41/4 weighted average before the course to 1.65/4 after the course).

**Conclusion:**

Our experiences serve as a valuable resource for educators seeking to implement similar improvement approaches in their educational settings. Furthermore, our findings offer insights into tailoring course development and teaching strategies to optimize student learning.

## Introduction

It is imperative for graduate students in biomedical, clinical, or translational science programs be equipped to design robust and responsible research studies and apply appropriate statistics that will be used to analyze, report, and interpret their data. It is only through such a foundational understanding of statistics that research findings can be effectively translated into clinical practice or leveraged to enact policy change. Despite the pivotal role of a strong methodological and statistical foundation for graduate students, acquiring adequate knowledge and skills can be challenging, especially for those without prior experiences in statistics. This is especially true as they transition into intensive research-based graduate programs, which are inherently self-directed and often constrained by limited time and access to appropriate foundational learning opportunities [1,2]. Studies have shown that 80% of graduate students experience a high level of statistics anxiety, defined as “a state-anxiety reaction to any situation in which a student is confronted with statistics in any form and at any time” (Onwuegbuzie, DaRos, and Ryan, 1997, p. 28) [3–5]. Further, the literature increasingly reports the prevalence of statistical errors in manuscripts published in peer-reviewed journals [6,7]. These can contribute to poor reproducibility in scientific research, reduce quality of scientific research, and lead to misleading conclusions [8,9]. The value of integrating a foundational statistics course is multifaceted. It can serve to set emerging medical researchers on track for success by facilitating acquisition of the skills to design and conduct high-quality research using correct statistical approaches, to ultimately produce more impactful and robust conclusions from their scientific research. It also ensures consistency in students’ abilities and alleviates some pressure from supervisors to support students who may come into the graduate program with inadequate fundamentals.

Our institute is the largest graduate unit in the Faculty of Medicine at a major Canadian university with over 700 faculty members and 500 graduate students. The institute offers full-time, research-intensive programs for both master’s and doctoral students across four main training areas: biomedical science, clinical science, health systems and services, and population health. Each stream offers diverse multidisciplinary fields of study, such as cardiovascular sciences, neuroscience, bioethics, membrane biology, respiratory medicine, transplantation, and psychosomatic medicine. The institute is committed to becoming a global leader in graduate education to improve human health through translational research. In all academic disciplines, the shared requirement among its students is that they must possess a strong foundation in study design and statistical methods to rigorously collect, evaluate, and interpret their data, which can ultimately help to advance scientific knowledge and improve healthcare outcomes.

In this study, we describe the use of the analyze, design, develop, implement, and evaluate (ADDIE) model [10] to create an introductory statistics and study design course for graduate students in the institute. We further demonstrate how the ADDIE model can be used iteratively, incorporating evaluation findings to inform and implement course refinements, ultimately improving the overall learning experience in statistics and study design for students.

## Approach: the ADDIE model

**Figure 1.**
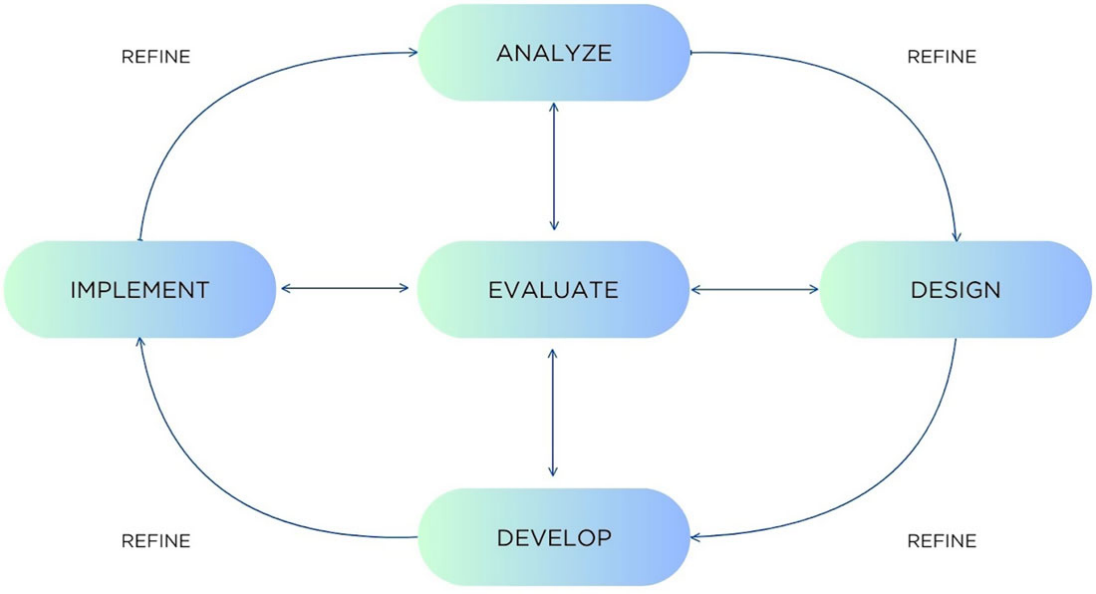
The analyze, design, develop, implement, and evaluate (ADDIE) model. Image adapted from Kurt 2017.

Many curriculum design models exist and are used frequently in post-secondary education [11]. Of the wide variety available, this work employed the ADDIE model [12,13] (Fig. 1) because of its iterative nature and its alignment with the ethos of graduate-level research to gather data, formulate actions based on the findings, implement an action plan, and study the results. Although it was originally designed to be used in a linear fashion [13] and was specifically designed for design and development of military training processes [12], revisions have created an updated model that is more flexible and fluid [14] and is more iterative and dynamic, situating evaluation across the processes instead of at a single phase [12]. It is comprised of five key steps that can facilitate a structured approach to instructional design, and when used iteratively, they can create a continuous quality improvement cycle for ongoing improvement of curriculum and its impact on student learning (Fig. 1) [15].

1. **Analyze**: gather information to ascertain the curricular issue
2. **Design**: use information to imagine how to meet the needs identified
3. **Develop**: plan the detailed elements of the course/intervention
4. **Implement**: deliver the final course
5. **Evaluate**: use predetermined metrics to assess the success and impact of the course

While the phases are described in a linear fashion below, it is important to note that the use of the phases was fluid and continuously informed and occasionally impacted decisions made at other phases.

In the context of this work, the ADDIE model was employed to leverage the results of an external institute review to inform the instructional design of an introductory statistics and study design course within the institute. Evaluation of a pilot offering of the newly developed course was then taken into consideration in making refinements for future iterations. The development workflow undertaken over a three-year timeframe is reported below, incorporating iterative learnings and modifications to provide a comprehensive picture of the ADDIE model at work. The Research Ethics Board at the institution approved this study (00045806).

## Intervention: course development and pilot evaluation

### Analyze: identifying curricular gaps

In 2018, the institute underwent an external review that led to several recommendations related to the curriculum, specifically highlighting the need for improved access to statistical data analysis content for students. To address this recommendation, an internal curriculum review was conducted between 2020 and 2021 using mixed methods, such as surveys, interviews, and focus groups, to identify curricular gaps and needs. Data from stakeholder surveys showed that 60% of students (78/130) perceived the need for courses that advanced their practical skills, particularly in statistics (44%, 57/130), while 51% of alumni (36/71) expressed a desire to have acquired enhanced statistical analysis skills during their time at the institute (Table 1). Eighty percent of supervisors (66/83) affirmed the importance of statistical and research methodological skills (Table 1). Results from in-depth interviews with supervisors across disciplines (basic science (*n*= 4), translational research (*n* = 2), and clinical research (*n*= 5)) emphasized the pressing need for improved access to content in statistics and research methodology to better equip students for research competence. Collectively, these findings highlighted the importance of developing a dedicated introductory statistics and study design course to be offered by the institute to improve access to the content for its graduate students.

**Table 1.** Stakeholder surveys to identify curricular gaps

Student survey (n = 130)	
Perceived need for course and program	Percentage (%)
Courses that advance their practical skills, particularly statistics, coding, and grant writing	60
Professional development support	38

Alumni survey (n = 71)	
Perceived gaps in curriculum/programming	Percentage (%)
Writing (thesis, manuscript, grant)	54
Statistical analysis	51
Critical thinking	50
Teaching	44
Communication	43
Method techniques	40
Coding	39
Conflict resolution	33
Other	6

Supervisor survey (n = 83)	
Perceived area for student development and competency	Percentage (%)
Statistical and research methodological skills	80
Grant and manuscript writing	60
Writing/presentation skills, general/specific knowledge, analytical skills	50
Professional development and networking skills	26

### Design: building the course framework

Once the recommendations of the curriculum review were accepted, a working group of the institute’s Curriculum Committee was struck to address the need for core study design and statistics training. The working group included two subject matter experts (one statistician and one computer science expert), one curriculum designer, one current senior PhD student, and one PhD alumnus. The two subject matter experts and curriculum designer were all faculty members on the Curriculum Committee. Course goals were drafted by the working group and reviewed and ratified by the broader Curriculum Committee. Final course goals were to provide students with the ability to understand and interpret statistics, enabling them to (1) conduct their own research and (2) critically appraise research evidence from the scientific and medical literature. Further, after review of the landscape of guiding principles and core topics for statistical training, and based on expert opinion from the working group, the revised Guidelines for Assessment and Instruction in Statistics Education (GAISE) recommendations [16], and the problem, plan, data, analysis, and conclusion (PPDAC) model [17] were selected to form the basis of the course structure. Oster and Enders identified a set of 24 statistical competencies for graduate students in clinical and translational science [1,2], which are suggested to determine topics that should be taught in statistical education and guide the overall design of the curriculum for students in clinical and translational science [1]. The revised GAISE recommendations [16], developed by the American Statistical Association to provide guidance on developing statistics education, are well aligned with statistical competencies work. The six GAISE recommendations include (1) teach statistical thinking (statistical literacy), (2) focus on conceptual understanding, (3) integrate real data with a context and purpose, (4) foster active learning, (5) use technology to explore concepts and analyze data, and (6) use assessments to improve and evaluate student learning [16]. In addition, two emphases for Recommendation 1 include: (1) teach statistics as an investigative process of problem-solving and decision-making, and (2) give students experience with multivariable thinking [16]. In a previous study comparing two teaching methods – flipped classroom using the GAISE recommendations and traditional lectures in an introductory statistics course – students in the flipped classroom using the GAISE recommendations demonstrated better performance in developing statistical literacy and more positive statistical perception than the students in traditional lectures [18]. Thus, we decided to use the GAISE recommendations with an emphasis on practical application of statistics using real-world data. In addition, we employed the PPDAC model to drive curricular flow, as mentioned in the GAISE recommendations [16,17]. The PPDAC model follows five stages: (1) outline the problem and define a research question to solve this problem, (2) plan a research study to answer the research question, (3) collect data, (4) analyze the study results using appropriate statistical methods, and (5) provide conclusions that reflect research findings [17]. This model was thought to be a solid methodological framework that would serve students well into their future research careers.

In addition, R with R studio was chosen as the statistical software because it is free, open source, and easily facilitates reproducibility of an analysis with tools such as R Markdown [19]. Finally, a “backward design method” [20] was used to guide instructional design, including articulating detailed learning objectives and aligned learning experiences and determining valid student assessment methodologies to show achievement of those goals.

Delivery format was also considered during the design phase. At this time, the majority of courses in the institute had been converted to online delivery due to COVID-19, and as such, both students and faculty had built up a level of comfort and competence with this format. Advantages and disadvantages of online delivery were weighed, and a decision to pilot this course with an online delivery format was made. The clear advantages were seen to be the accessibility for a student population that was situated in research labs across a wide geographical area, and the perceived ease to scale the course up if demand increased.

### Develop: establish the course content

Once goals and objectives were established, the detailed course was built. The online, synchronous course was structured for 12 weeks including weekly two-hour lectures and one-hour tutorials. Lecture content was selected to align with the PPDAC model and was to be delivered by subject matter experts on specific topics (Table 2). Tutorials provide students with opportunities to integrate and consolidate information; to apply statistical knowledge gained during the lectures in contextualized scenarios; and to facilitate practical application of R software for the analysis techniques discussed in the lectures. Student assessment methods were chosen to motivate students to keep up with the course content through weekly quizzes. A midterm test was added to gauge students’ progress early enough in the course to identify issues, and a final project was chosen to allow students to demonstrate their comprehension of the material.

**Table 2.** Schematic outline of course elements

Week	Lecture Topic	Tutorials	Course Work	P	P	D	A	C
1	Introduction – Fundamental concepts in statistics Populations and samples; parameters and statistics; bias and sampling variability; descriptive statistics and inference; variability and uncertainty; random variables; probability distribution and sampling distributions Introduction to PPDAC	Tutorial 1		X	X	X	X	X
2	Research Design I – Important Design Issues Formulating the research question Sample size and power Choosing primary and secondary outcomes Who is your target population? Sampling – how do you choose your subjects?	Tutorial 2	Online Quiz 1	X	X			
3	Research Design II – Stats and Protocol Writing Common statistical designs in medical research Writing a research protocol: SPIRIT statement	Tutorial 3			X			
4	Data Collection/Management Deciding on what data to collect Designing and testing data collection instruments Designing questionnaires Questionnaire measurement scales Data entry, Data entry checks	Tutorial 4	Online Quiz 2			X		
5	Displaying/Summarizing Data	Tutorial 5					X	
6			Project Proposal Presentations					
7	Comparing Two or More Groups with Continuous Data, Comparing Groups of Binary and Categorical Data	Tutorial 6	Online Quiz 3				X	
8	Correlation, Linear Regression	Tutorial 7					X	
9	Logistic regression	Tutorial 8	Online Quiz 4				X	
10	Missing Data The problem of missing data Strategies to minimize missing data Types of missing data Analysis methods to deal with missing data	Tutorial 9	Critique Assignment		X		X	
11	Reporting guidelines for research findings CONSORT statement and checklist for randomized controlled trials STROBE statement and checklist for observational studies	Tutorial 10	Online Quiz 5					X
12	Presenting research findings ICMJE criteria for authorship Presenting statistics in research articles Numerical results P values and confidence intervals Tables and graphs Statistician’s checklist Common causes for rejection of medical papers based on a statistical review							X
13			Final Project					

CONSORT = Consolidated Standards of Reporting Trials.

ICMJE = International Committee of Medical Journal Editors.

PPDAC = Problem, Plan, Data, Analysis and Conclusion.

SPIRIT = Standard Protocol Items: Recommendations for Interventional Trials.

STROBE = Strengthening the Reporting of Observational Studies in Epidemiology.

### Implement: piloting the course

The course, entitled “Learning from Data – An Introduction to Study Design and Statistical Analysis Methods,” was delivered for the first time in the Fall 2021 semester (September to December). Seventy-three students registered for the course. Fifty students completed the course delivered by four teaching assistants (TAs), five course lecturers, and a course director. Twenty-three students (31.5%) withdrew from the course before its completion.

### Evaluate: assessing the pilot

A rigorous evaluation scheme was created to monitor the outcomes of the course using several evaluation tools. An anonymous, 16-item online course evaluation survey was distributed at the end of the course to all students who completed the course. An anonymous course withdrawal survey was distributed to the 23 students who withdrew from the course to identify reasons for withdrawing from the course. Two focus groups were conducted – one with course faculty and TAs and another with student representatives from the institute’s Students’ Association.

#### Course evaluation survey

**Figure 2.**
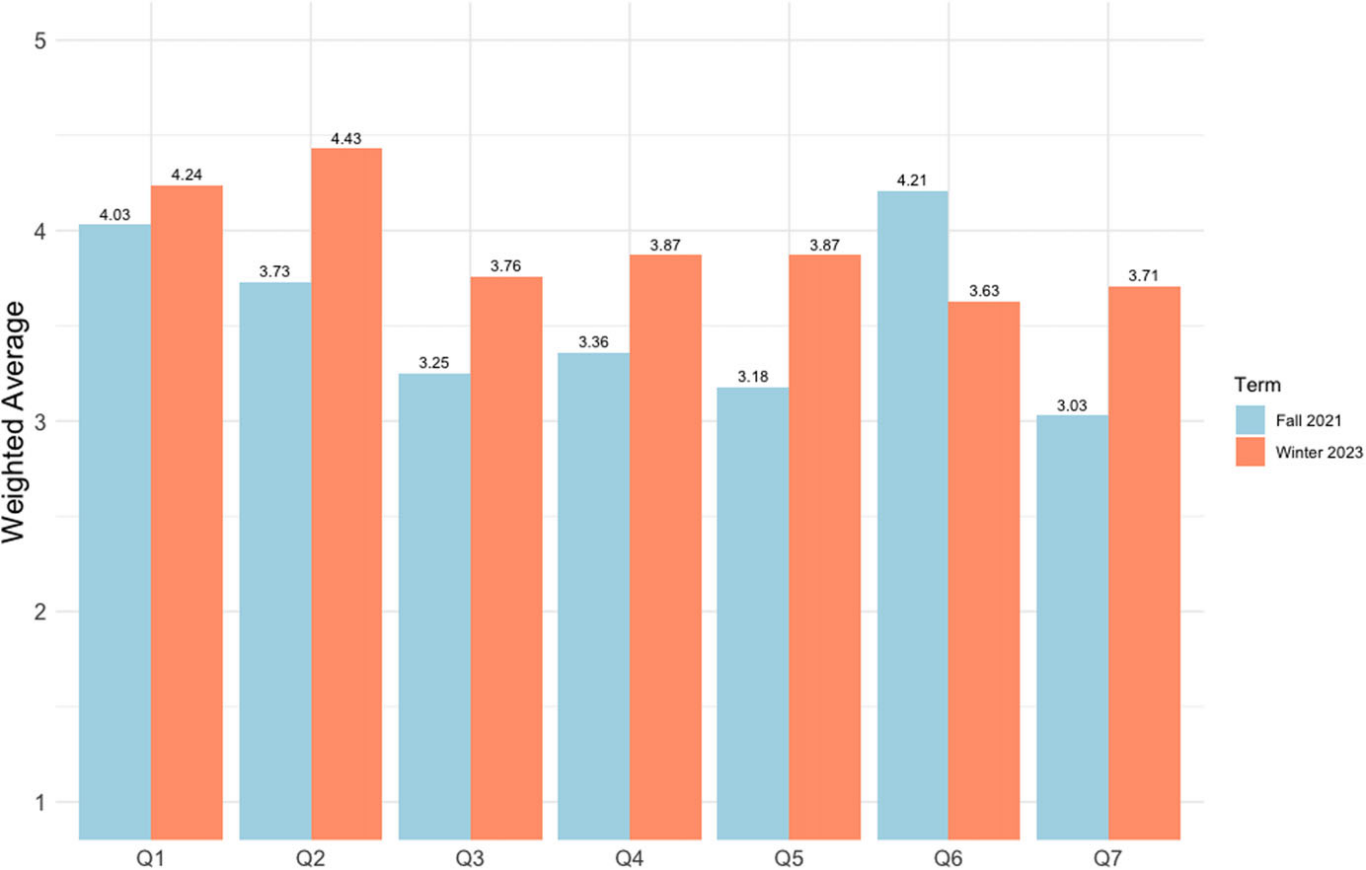
Student course evaluation survey in Fall 2021 (n = 33) and Winter 2023 (n = 38). Full question from left to right: Q1: “I found the course intellectually stimulating.” (1 = Not at all, 5 = A great deal); Q2: “The course provided me with a deeper understanding of the subject matter.” (1 = Not at all, 5 = A great deal); Q3: “Course projects, assignments, tests, and/or exams improved my understanding of the course material.” (1 = Not at all, 5 = A great deal); Q4: “Course projects, assignments, tests, and/or exams provided an opportunity for me to demonstrate an understanding of the course material.” (1 = Not at all, 5 = A great deal); Q5: “Overall, the quality of my learning experience in this course was:” (1 = Poor, 5 = Excellent); Q6: “Compared to other courses, the workload for this course was:” (1 = Very light, 5 = Very heavy); Q7: “I would recommend this course to other students.” (1 = Not at all, 5 = Strongly).

Thirty-three students (66%) completed the survey (Fig. 2). The highest rated elements (weighted average; 1 = not at all, 5 = a great deal) in the course evaluation survey were, “I found the course intellectually stimulating” (4.03/5) and “the course provided me with a deeper understanding of the subject matter” (3.73/5). The least favoured elements were, “I would recommend this course to other students” (3.03/5) and “compared to other courses, the workload for this course was…” (4.21/5; 1 = very light, 5 = very heavy). Students that completed the course were asked to provide open-ended feedback (n = 23, Table 3). Notable themes that emerged from these comments were related to heavy workload (5/23, 22%) and unclear instructions or too much content in the course lectures and evaluation items (12/23, 52%).

**Table 3.** Examples of student feedback in Fall 2021 and Winter 2023

Fall 2021	Winter 2023
“Structure of the course felt like an undergraduate course, not suitable for a graduate course.”	“The changes [the course director] implemented based on last year’s student feedback really improved the course.”
“Time spent on this course was nearly triple of other graduate courses and significantly took away thesis writing time.”	“Very useful content for my thesis project, a great environment to learn and a very manageable amount of work.”
“Assignment instructions and expectations were not clear.”	“The instructions were clear and engaging. Overall, the quality of delivery and instruction of course material was very good.”
“Weekly quizzes were poorly structured and too much to complete within the given timeframe.”	“The presentation was a good idea to provide students with an opportunity to practice public speaking and science communication – a practical skill that will be useful within our own research.”
There was a “disconnect between teacher and learner expectations.”	“Instructors did a great job at making the concepts and materials easy to follow and understand.”

#### Course withdrawal survey

Nine students (39%) completed the course withdrawal survey (Table 4). More than half of the students indicated a heavy workload (5/9, 56%) as the reason for withdrawing from the course. Other reasons included a lack of coding experience, the theoretical nature of the lecture content, a heavy workload needing memorization, excessive course evaluation items, concerns about time commitments, and later realizing that the course was not needed to fulfill the student’s program requirements.

**Table 4.** Students’ reasons for withdrawing from the course in Fall 2021 (n = 9)

Reasons for withdrawing from the course	n
Heavy workload	5
Did not like teaching style	5
Did not like the evaluation methods of the course	4
Format was not conducive to learning	4
Content was familiar/repetitive	2
Course was not applicable to research	1
Topic of the course was not as described	1
Course was unorganized	1
Not enough background to take the course	1
Other	6

#### Faculty and students’ association focus groups

A focus group discussion with the course director and TAs similarly noted workload as the primary area requiring attention. Other areas identified included simplifying the lectures by reducing technical content, minimizing the use of statistical jargon and unfamiliar language for students, and extending the time allotted for completing quizzes. The other focus group with student representatives yielded additional suggestions including substituting the written midterm exam with an oral presentation to demonstrate statistical knowledge that would emphasize the value of developing research-related presentation skills over exam-writing proficiency. The second suggestion was to offer an opportunity for students to apply the study design and statistical analysis methods learned in the course to their individual, real-world research datasets.

### Second ADDIE iteration

Following the initial pilot and evaluation of the “Learning from Data” course in the Fall of 2021, modifications were considered and implemented prior to a second iteration of the course in the Winter of 2023 (January to April). Incorporation of feedback required a second pass through the ADDIE model, highlighting the value of such a structured and systematic approach. When making refinements, as opposed to informing the initial development of a course, it seemed to be appropriate to collapse certain steps and to consider things in a more interrelated manner, and the second pass at ADDIE is thus reported in this single section, reflecting the refinement work done in advance of (and following) a second offering of the course, in the Winter of 2023.

Based on the multifaceted evaluation from the Fall 2021 course offering (linking the Evaluate step to an iterative consideration of the Analyze reflection on curricular gaps), a number of suggested refinements were incorporated for Winter 2023. Refinements reflected a revisitation of the Design and Develop steps. The majority of these related to the structure and contextualization of the course (Design), rather than the content (Develop).

“Design”

- Increase the visibility and engagement of the course director
- Move the course to the winter semester to allow students to integrate into their programs and labs before taking this course
- Reduce the frequency of weekly knowledge quizzes to bi-weekly
- Replace the written midterm exam with presentations
- Build and implement a clearer evaluation rubric for the course project

“Develop”

- Make minor modification to course content to reduce workload
- Allow students to use their own, real-world data to improve relevance of the course
- Make the textbook readings supplementary instead of mandatory
- Convert the focus of quizzes to the lecture material instead of the reading material

For Winter 2023, all the same evaluation methods were employed. In addition, a survey on students’ self-perceived level of statistics anxiety was administered once at the end of the course, asking them to reflect on their statistics anxiety levels before and after completing the course. Fifty-three students completed the second iteration of the course. Seven students (11.7%) withdrew from the course. Between Fall 2021 and Winter 2023, overall institute course withdrawal rates ranged from 0 to 44%, with an average of 12%. The first iteration of this course had a withdrawal rate at the high end of this range (31.5%), while the second iteration was closer to the average. The second iteration of the course was delivered by six TAs (three from original iteration), three course lecturers (who also taught in the first iteration), and the same course director. Evaluation demonstrated that the course modifications led to several measurable improvements. In this paper, we report on the comparison of the course evaluation survey results from the first to second iteration and on the findings from the statistics anxiety assessment.

#### Course evaluation survey

Data from the course evaluation survey demonstrated that the student experience consistently improved with the most notable improvements seen for the following statements (1 = not at all, 5 = a great deal) from Fall 2021 to Winter 2023: (1) “the course provided me with deeper understanding of the subject matter” from 3.73/5 to 4.43/5 (+0.70/5), (2) “overall, the quality of my learning experience in this course was…” from 3.18/5 to 3.87/5 (+0.69/5), and (3) “I would recommend this course to others” from 3.03/5 to 3.71/5 (+0.68/5) (Fig. 2). Generally, the majority of open-ended student feedback in Winter 2023 was notably positive, in contrast to Fall 2021 when the majority of feedback focused on suggestions for future improvements (Table 3). However, some students still suggested that the time allotted for completing quizzes should be extended (3/30, 10%). Across both iterations of the course, students particularly appreciated the tutorials and the support provided by the TAs.

#### Statistics anxiety survey (New for 2023)

**Figure 3.**
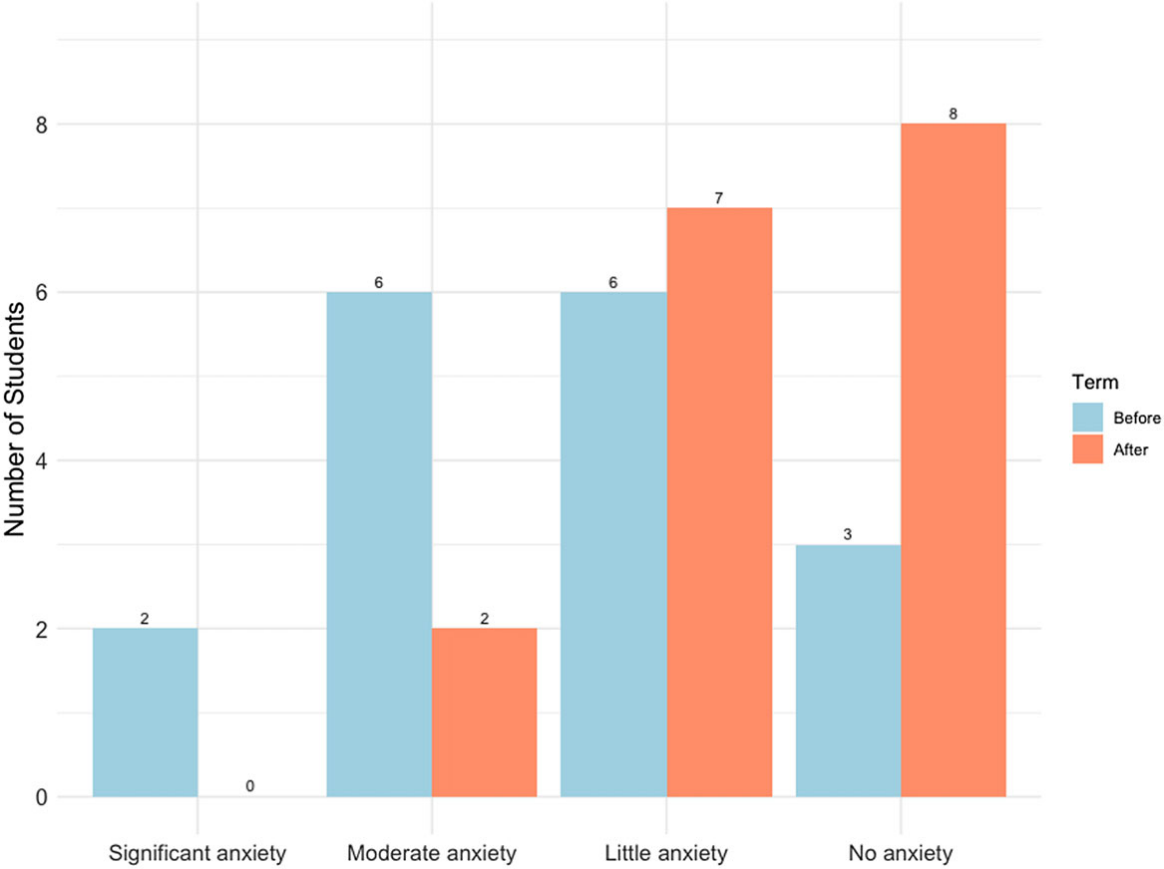
Self-perceived level of statistics anxiety before and after taking the course in Winter 2023 (n = 17).

Results from the statistics anxiety survey (n = 17) showed that students’ perceived level of statistics anxiety decreased from 2.41/4 (weighted average, 1 = no anxiety, 4 = great anxiety) before the course to 1.65/4 after the course (−0.76/4) (Table 5, Fig. 3). Of these students, 16/17 (94%) agreed that the course helped them to feel better about statistical analysis. One student that disagreed used online resources to help them feel better about doing statistical analysis.

**Table 5.** Questions from the statistics anxiety survey

Question	Response Options
**Q1**: How did you feel about taking a statistics course or doing statistical analysis before the course?	Likert Scale: 1 (No anxiety) to 4 (Considerable Anxiety)
**Q2**: How did you feel about taking a statistics course or doing statistical analysis after the course?	Likert Scale: 1 (No anxiety) to 4 (Considerable Anxiety)
**Q3**: Did the course help you feel better about doing statistical analysis?	Yes, No, Not Applicable
**Q4**: What other resources did you access to help you feel better about doing statistical analysis? (If answered “no” above)	Dropdown Menu: Private Tutor, Peers, Online Resources, None, Other

## Discussion

Very little has been written about deliberate approaches to curriculum design for introductory statistics courses in graduate programs with a research focus despite the compelling rationale to ensure adequate study design and statistical methods training. It is possible that the curricular content required is felt to be so niche that faculty often forego the more established approaches to curriculum design and evaluation used in undergraduate education or in graduate programs with more structured course work. Thus, effective course design and teaching methods for introductory statistics education in research-based graduate programs remain underexplored and underreported. The importance of the skills and the gap in literature underscore the need for educators to share their experiences and tools, including the challenges and lessons learned, to provide valuable insights to other educators in similar educational settings seeking to integrate statistical education into their graduate programs. This was echoed in the original curriculum review where 44% of students who responded to the needs assessment survey indicated that they require additional training in statistics and 51% of alumni respondents identified that they wish they had acquired more advanced preparation in study design and statistics.

Curriculum reformation in biomedical and clinical graduate research programs can be additionally difficult given that courses often originate in discipline-specific departments that were traditionally siloed and thus maintain that legacy of narrow focus [21] and that significant resources must be marshaled to support any curricular change process [22]. While few papers address formal curriculum design and development in graduate programs [21,22], it is seen to be of ever increasing importance in the face of changes like the “data deluge” [23] and the emergence of data science and analytics as disciplines. The ADDIE model, and its simple and flexible approach, was useful in breaking down traditional assumptions and permitted a holistic approach to articulating the fundamentals of good study design and the selection of appropriate statistical methods that will permit research findings to be effectively translated into clinical practice or leveraged to enact policy change.

Of course, any curricular changes should be accompanied by robust course evaluation methods. While the ADDIE model articulates evaluation as a discrete phase, newer versions of the model emphasize evaluation and feedback be embedded at all phases and feed the iterative use of the model. A variety of methods can be used to gather valuable information about the quality of a course depending on the level of impact being considered. Kirkpatrick’s hierarchy of outcomes sets out four levels of impact, all of which should be considered during course evaluation [24]. While we implemented a variety of tools to evaluate various levels of impact, this work addresses our measurement of level 1 and 2 impacts (reaction and learning) [24]. Results revealed consistently higher levels of satisfaction with the second iteration of the course, and it is hoped that ongoing use of the model and evaluation tools will permit additional gains in future iterations.

We were also interested in the concept of “statistics anxiety,” which is being increasingly studied and reported on in the literature. In one study, researchers examined doctoral students in an educational technology program, who took an online statistics course [25]. While they reported on the instructional elements of the course that were most and least effective and liked by the students, they also reported that statistics anxiety [3,4], specifically test and class anxiety (anxiety related to students taking the statistics course and exams), interpretation anxiety (anxiety when interpreting or making decisions using statistical results), and computation anxiety (anxiety toward mathematical equations and calculations of statistics), decreased over the course of the semester [25]. As an emerging trend with graduate students, this study attempted to understand how, if at all, the completion of the course impacted the learners’ level of statistics anxiety. Ongoing revisions to the course will attempt to address this prevalent challenge for our students.

A key strength of our study is the longitudinal and systematic nature of our assessment of the effectiveness of the course. The cycle of implementing, evaluating, and acting on findings is implemented across all courses in our institute and embedded in our curriculum review cycle. The routine and perpetual nature of the activity makes it easier to facilitate the process and monitor the ongoing effectiveness of the process.

Of course, there are challenges inherent in the use of a structured model for instructional design of a new course. First, it is challenging to systematically gather large amounts of data from various stakeholders and implement notable refinements to a course. This demands both time and resources for the original development and subsequently an administrative infrastructure and dedicated support to execute systematically and effectively over time.

There are limitations to this study. This was an exploratory study conducted with a relatively small sample size in two iterations of the course and while the total number of students might be relatively low, this course is still one of the largest in our institute. Nevertheless, the sample size included in this study is reflective of a standard graduate course size, and we were able to demonstrate measurable improvements over time. Continued course evaluations and refinements to the course are critical to better understand the impact of the course and its adaptability to other programs, but our results must be interpreted with caution when generalizing to other programs and student populations.

Another limitation of our study relates to the survey about the self-perceived level of statistics anxiety. The simple, four-question survey (with a 4-point Likert scale) was administered one-time only, at the end of the course, which could have resulted in recall bias [26]. This was done to maximize student participation in this voluntary survey, ensuring minimal time pressure and inconvenience. While valuable insights were derived from analyzing this data, in the future, the validated STARS survey [4,27,28] will be employed at the beginning and end of the course to reduce potential recall bias. In-depth understanding of statistics anxiety would help course instructors to optimize teaching strategies that minimize stress and enhance overall learning experience for students.

Finally, there was low completion rate of course evaluation surveys (66% in Fall 2021, 72% in Winter 2023) and the statistics anxiety survey (32%). Students with higher anxiety and lower engagement may be less likely to complete the surveys, which may have led to an overestimation of effects; therefore, the results must be interpreted with caution. Past studies have also shown that students were more likely to participate in student course evaluation surveys when they felt assured about retaining anonymity, as they were concerned about potential academic repercussions if identified [29,30]. Nair et al reported other factors including survey length, timing, engagement of students, use of multiple contacts, and offering incentives [31–35]. In addition, online responses typically elicit lower response rates than in-class administration of surveys [33,34]. All of these factors play a role in the quality and quantity of information gathered and must be triangulated with other data sources.

## Conclusion

The findings of this study demonstrate the value in a systematic approach to considering and addressing foundational learning needs in a graduate department. Upon identifying the need for introductory statistics and study design competence across the graduate student population, the department was well-served by an evidence-based approach to informing a novel course. Subsequent piloting and refinement led to notable improvements in students’ learning experience, as well as a positive shift in attitudes and perceptions toward statistics after students completed the course. Such an approach has the potential to be applied in similar education settings where similar needs exist for cohesive, effective training in statistics and potentially in other foundational content identified through an evidence-based process.
